# User engagement in mobile apps for people with schizophrenia: A scoping review

**DOI:** 10.3389/fdgth.2022.1023592

**Published:** 2023-01-10

**Authors:** Raquel Simões de Almeida, António Marques

**Affiliations:** Center for Rehabilitation Research, School of Health, Polytechnic of Porto, Porto, Portugal

**Keywords:** engagement, schizophrenia, mHealth, mobile app, scoping review

## Abstract

Over the past decade, there has been an increase in the number of mobile apps designed for mental health proposes and mHealth has been perceived as a promising approach to help people with schizophrenia to manage their condition. However, adoption rates are relatively low and long-term user engagement is a major issue. The aim of this study is to identify and better understand what strategies and factors may influence user engagement and facilitate prolonged use of apps for people with schizophrenia to better manage their illness. A scoping review was conducted in accordance with the Arksey and O’Malley scoping review framework and following PRISMA ScR guidelines. The sources consisted of searching four electronic databases. Rayyan software was used for this study selection process and a narrative approach was used to synthesize the extracted data. A total of 28 studies which met the inclusion criteria were identified. The engagement strategies included push notifications, message prompts, personalization, application customization, goal setting, game-like features, use of different multimedia formats, social connectedness, support (peers and professionals), reliability of content and quality of feedback received. Some demographic factors may influence adherence such as age, gender, education level and socioeconomic status. Other factors also may play a role impacting engagement: health status, data privacy and security, involvement in design process, incentives for participation, app usage fitting in the user routines, initial training, and constant technical support. Included studies present high heterogeneity in outcome measures and thresholds criteria to assess engagement. Understanding what influences engagement and how to measure it is essential to enhance the design of mobile apps and deliver scalable solutions to help people with schizophrenia better manage their illness in their real-world uptake.

## Introduction

The growing prevalence of digital technologies in healthcare has created great interest in mHealth in the Psychiatry field. With the constant development of smartphones, mobile applications (apps) have become important tools for assisting with the diagnosis, educating, monitoring, treating, and encouraging people to manage their mental health problems. Besides providing a unique opportunity to expand the availability of mental health assessments and interventions, apps could enhance the quality of the services delivered. This is important since the contemporary paradigm determines that people with mental health problems, especially those with schizophrenia, could have access to digital training and take more responsibility for their own care collaborating with clinicians to attain their personally defined and valued goals, using their self-determination, opposing the high chronicity, disability, and the burden continuously associated with this illness (1).

Regarding technology use, several studies found that mobile phone ownership among patients with schizophrenia is high, around 80%, and that the majority owned a smartphone (1–5). Younger patients have fewer negative symptoms and better psychosocial functionality and were likely to use technological devices more (6). Moreover, among people diagnosed with schizophrenia, the willingness to use smartphone apps is associated not only with age, but also with education, income, and device type (7). Also, some mental health practitioners are apprehensive that psychotic symptoms such as paranoid delusions may limit the feasibility of mobile health interventions. However, several studies have shown that mobile interventions are a viable strategy for people with psychosis, and they express their satisfaction with these interventions, finding them useful, beneficial, and easy to use, not triggering a worsening of symptoms (8, 9). Still, patients with more adverse symptoms or more severe intellectual deficits will struggle to use apps and could benefit more with other interventions.

However, high interest in mobile apps for mental health does not automatically mean high involvement in using them over time which affects the effectiveness of mobile interventions (10–12). Mental health apps have high dropout rates and a very low user engagement over time, not only in clinical trials but also in the real-world (13).

Some authors argue that this situation is due to negative or positive symptomatology or cognitive disfunction (3, 14), however it could also be cause by a poor user experience and usability. A study from Alqahtani and Orji (15) said that poor usability, lack of a content variety and personalization, lack of customer service, and security and privacy issues are the most common reason for abandoning mental health apps. Other authors claim that user experience does not predict sustained engagement with mental health apps and that it is necessary to define what sustained engagement means in this scope, what leads to it, and how to create products that achieve it (16). There are several engagement definitions (17) and defining meaningful engagement for digital mental health interventions remains a challenge as well as comparing engagement across studies or tools (18). A good example of engagement definition within digital interventions can be translated as “the extent (e.g., amount, frequency, duration, depth) of usage and a subjective experience characterized by attention, interest and affect” (19). Being a multidimensional concept, critical for mHealth effectiveness, it is essential to have a clear understanding of which user engagement indicators to report, namely “usability,” “user satisfaction”, “acceptability”, and “feasibility”. Moreover, there are subjective and objective criteria used to assess user engagement indicators, and behavioral measures tend to focus more on product metrics rather than in the recovery process success.

The definition of success regarding user engagement, means different things to different audiences. For instance, for a patient experiencing acute symptoms, the simple act of logging in the app and asking for support can be seen as a successful experience. For other users that may benefit from regular and repeated interaction with an app, success may be defined by the number and the quality of interactions between the user and the app. Both scenarios have very different definitions of success (20).

There are several models and theories that study engagement strategies. One of these models is the Persuasive System Design (PSD), a framework that defines four different mechanisms to increase user engagement by (1) focus on the central purpose of the app, (2) promoting user-app interactions, (3) foster social relationships between users and other profiles, and (4) increasing app credibility (21). Some mHealth apps use game-design elements to foment and maintain health-behavior outcomes. Although evidence suggests that gamification can boost health-behavioral outcomes, the results are not consistent and does not guarantee long-term use and adherence to mHealth (22).

Some authors argue that strategies complementary to the app's core feature such as reminders, feedback, coaching and peer support, tend to increase user engagement. Still, the absence of a common criteria that can measure engagement and usability across studies, makes it harder to distinguish which of the studies around the subject really bring some improvement on user engagement, regardless of the common claims of success by most of these works (18).

Despite the promise made by some mHealth apps for people with schizophrenia that enable them to self-manage their illness, adoption rates are relatively low and long-term user engagement is still an issue. Thus, in this scoping review we aim to identify:

(1) What strategies are applied to improve user engagement with mobile apps for schizophrenia self-management?
(2) What factors result in better user engagement for people with schizophrenia?
(3) How user engagement is being measured?

## Materials and methods

This scoping review was conducted in accordance with the five-stage process outlined by Arksey and O'Malley (23) identifying the research question, identifying relevant studies, study selection, charting the data, and collating, summarizing, and reporting the results and is reported in line with PRISMA-ScR (Preferred Reporting Items for Systematic Reviews and Meta-Analyses extension for Scoping Reviews) guidelines (24) reporting guidance. The protocol was made available to the Open Science Framework (https://osf.io/awh3z/) in May 2022.

### Search strategy

Systematic search queries of *Web of Science*, *PubMed*, *Academic Search Complete* and *IEEE Explore* were used to identify references published or available online in the last 10 years, to avoid discussing mobile apps that are potentially out of date. This search was done from April to June 2022 and all types of primary peer-reviewed research papers were considered. A sample search strategy comprised the terms: (“schizo*” OR “psychosis” OR “psychotic disorder*”) AND (“self-management” OR “self-help”) AND (“mhealth” OR “app” OR “smartphone”). The chosen keywords were selected based on the literature and the specific aim of our study.

### Eligibility criteria

After the records obtained were imported onto Rayyan (25), which is an online platform designed for multiple reviewers to work on systematic reviews, titles and abstracts were reviewed for eligibility. Reviewers are kept blind to each other's decisions, and can mark records as “include,” “exclude,” or “maybe” and can also mark exclusion reasons or add notes. This process was used to determine which records would be brought forward to full text review.

The PCC framework (Population or Participants)/Concept/Context) was used to guide study selection and to align the eligibility criteria with the research questions. Accordingly, to meet the eligibility criteria, the included papers had to (1) address a mobile app (programs or software applications designed to run on a mobile device such as a smartphone), (2) be designed for patients with schizophrenia spectrum disorders, (3) to manage their illness (including interventions such as relapse prevention, adherence to medications and/or treatment, psychoeducation, symptom monitoring) and (4) be published in English. Only apps designed specifically for people with schizophrenia spectrum disorders will be included, not general mental health apps used by those with these conditions since the purpose is to study how the applications for this population should be designed considering their characteristics. These papers should report on some aspects of user experience, using specific concepts, namely “usability,” “user satisfaction”, “acceptability”, and “feasibility”. There were no restrictions on characteristics pertaining to participants (e.g., age, ethnicity, gender), population (e.g., adult, youth) or setting (e.g., clinical, community).

The exclusion criteria were as follows:

- Studies without primary data, including reviews, commentaries, and study protocols.
- Studies that did not report engagement strategies in the intervention design.

### Study selection

Study selection was done in two steps. First, after duplicates were removed, the titles and abstracts of all retrieved articles were screened for eligibility by two authors (RSA and AM). Next, the full text of all remaining publications was checked for inclusion by the same authors. Disagreements on the inclusion or exclusion of publications were discussed until agreement was reached. The average percentage of agreement between authors was approximately 90%. Consistent with the PRISMA-ScR guidelines and the framework proposed by Arksey and O’Malley (23), a quality appraisal was not conducted.

### Data extraction

Data from eligible papers were extracted by one author (RSA) and checked for accuracy by the other author (AM). A data-charting form was developed and calibrated by the team. Data items that were extracted from each included study were authors names, country of origin, year of publication, study design and purpose, app name, type of engagement strategy, factors influencing engagement, used measures, and main findings.

## Results

**Figure 1:**
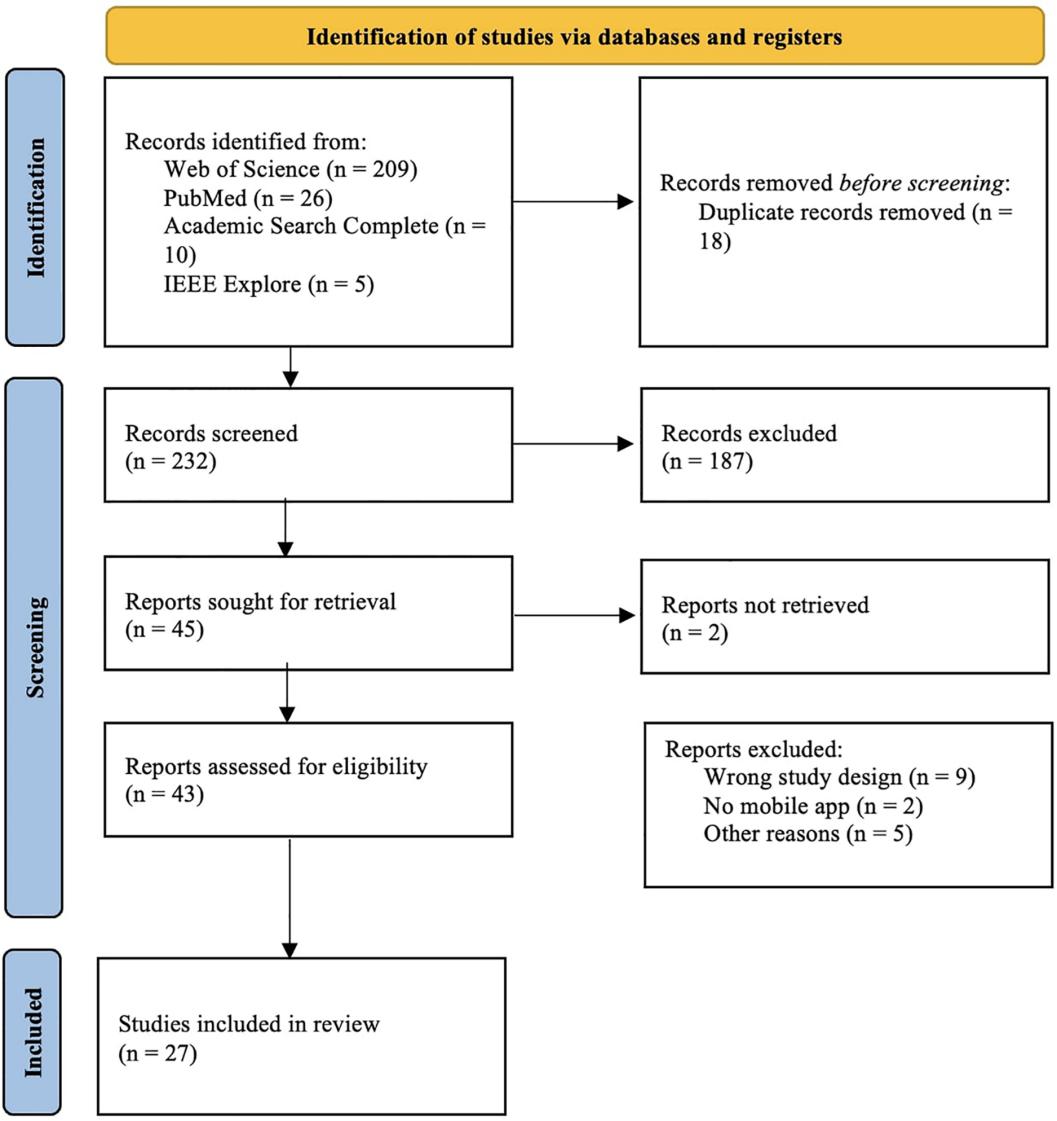
Selection of studies - PRISMA flowchart.

The search yielded 232 unique titles after duplicates were removed. After title and abstract analysis, 43 papers were assessed for eligibility and full text screening. In total, 27 papers published from 2012 up until 2022 were included (Figure 1).

### Study characteristics

**Figure 2:**
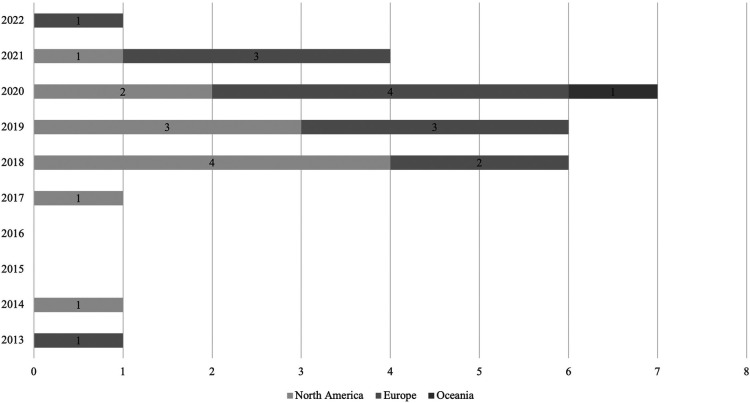
Number of included articles by year of publication and continent of authors.

Most of the publications emanated from the USA (*n* = 10), nine publications originated from UK, two from Canada, two from Australia and the others from other European countries (Denmark, Poland, Portugal, The Netherlands, Spain) (Figure 2). Mixed methods (*n* = 12) and quantitative studies (*n* = 11) were the most common data collection methods used, accounting for 82% (23/28) of all studies. Most samples used in these studies were relatively small ranging from 5 to 361 participants. Study duration (which in this article translates to the time app was used/tested) ranged from 6 days to 14 months.

Regarding included studies, some of them analyzed data from the same apps, however, we included them since the sample and the information retrieved were different. Apps like FOCUS (26–29), SMARTapp (30), MACS (31), weCope (32), My Journey 3 (33, 34), and App4Independence (35) offers illness management strategies in general. Others are more focused on symptom monitoring, namely Crosscheck (36), Actissist (37), ClinTouch (38), ExPRESS (39), Ginger.io (40), ReMindCare (41), and MindFrame (42). Other mobile applications are supported in CBT (SlowMo, 43; TechCareApp, 44; movisensXS, 45; PRIME, 46), or work as an agenda (MONEO, 47) or target specific domains such as social skills (MASS, 48), sleep (ExpiWell, 49), medication adherence (MedActive, 50), or delivers positive psychology content (+Connect, 51). Included studies are described in Table 1.

**Table 1:** Characteristics of included studies.

Authors/Year	Country	N	Study/Aim/Duration app use	App characteristics
Achtyes et al. 2019 (26)	USA	A total of 368 participants were enrolled in the study, of whom 356 used the app during the 6-month period. Due to some factors, 347 patients comprise the analysis set No data available about age mean and SD	Type of study: prospective, ten-site, longitudinal study of four technology-assisted interventions for patients with schizophrenia spectrum disorders within 60 days of psychiatric hospital discharge Aim: to better understand off-hours use of a smartphone intervention to extend support for people with schizophrenia spectrum disorders recently discharged from a psychiatric hospital Duration: 6 months	FOCUS: the app provided patients with access to illness management strategies in the areas of medication, mood, social, sleep and voices. That could be preprogrammed by the patient or be self-initiated by the patient on-demand when he/she felt the need for help, which could be used by patients as needed, providing help in five content areas: medications, mood, social, sleep and voices
Ben-Zeev et al. 2014 (27)	USA	33 participants (1 participant dropped out of the study after losing 2 study smartphones in the first week; the remaining 32 used the system throughout the trial, however 2 participants system use data were lost due to technical problems during the automated data transfer, therefore these reports concern 30 individuals) Mean age of 45.9 years (SD = 8.78)	Type of study: pre and post study trial Aim: to analyze feasibility, acceptability, and preliminary efficacy of the app Duration: 1 month	FOCUS: an app that offers users both prescheduled and on-demand resources to facilitate symptom management, mood regulation, medication adherence, social functioning, and improved sleep. The system is comprised of 3 applications that are installed onto the smartphone, and a web-based dashboard. The first app prompts users to engage daily *via* auditory signals and visual notifications that appear on the screen. The second is the primary FOCUS app that uses interactive algorithms to generate brief assessments and interventions that the user progresses through using touchscreen buttons on the smartphone home screen. The third is a Quick Tips app that allows users to access illness self-management resources and suggested coping strategies from a menu of options
Ben-Zeev et al. 2017 (36)	USA	5 participants Mean age of 26.4 years (SD = 7.33)	Type of study: randomized controlled trial Aim: to describe and demonstrate a novel app Duration: 12 months	Crosscheck: a multimodal data collection system designed to aid in continuous remote monitoring and identification of subjective and objective indicators of psychotic relapse. Besides gathering brief self-reports multiple times a week (ecological momentary assessment), multi-modal behavioral sensing (i.e., physical activity, geospatial activity, speech frequency and duration) and device use data (i.e., call and text activity, app use) are captured automatically
Ben-Zeev et al. 2018 (28)	USA	163 participants (82—FOCUS; 81—WRAP; 49% of diagnosis were schizophrenia or schizoaffective disorder) Mean age of 49 years (SD = 9.8)—FOCUS; Mean age of 49 years (SD = 10.1)—WRAP	Type of study: randomized controlled trial was conducted of a smartphone-delivered intervention (FOCUS) versus a clinic-based group intervention [Wellness Recovery Action Plan (WRAP)] Aim: to compare a mobile app (FOCUS) versus a clinic-based group intervention for people with serious mental illness Duration: 3 months (Interventions were deployed for a period of 12 weeks, using cycles of eight cohorts of participants assigned to individual FOCUS or group-based WRAP over parallel periods)	FOCUS: a multimodal, smartphone-delivered intervention for people with serious mental illness that includes three components: FOCUS application, clinician dashboard, and mHealth support specialist. The system includes preprogrammed daily self-assessment prompts and on-demand functions that can be accessed 24 h a day. Self-management content targets five broad domains: voices (coping with auditory hallucinations *via* cognitive restructuring, distraction, and guided hypothesis testing), mood (managing depression and anxiety *via* behavioral activation, relaxation techniques, and supportive content), sleep (sleep hygiene, relaxation, and health and wellness psychoeducation), social functioning (cognitive restructuring of persecutory ideation, anger management, activity scheduling, and skills training), and medication (behavioral tailoring, reminders, and psychoeducation)
Ben-Zeev et al. 2018 (29)	USA	9 participants used the intervention (1 participant dropout) Mean age of 45.5 years (SD = 13.18)	Type of study: mixed methods design Aim: to examine whether video-based mHealth interventions are feasible, acceptable, understandable, and engaging to people with schizophrenia Duration: 1 month	FOCUS-AV: a mobile system that offers video and written intervention options. FOCUS was designed for people with schizophrenia and offers both pre-scheduled and on-demand illness management interventions targeting auditory hallucinations, social functioning, medication use, mood problems, and sleep disturbances
Bonet et al. 2020 (41)	Spain	90 participants (59 participants used the app and 31 did not agree to use the app—treatment as usual group) Mean age of the sample was 32.8 years (SD = 9.4)	Type of study: usability study Aim: to assess the efficacy and clinical outcomes of the use of the app after 19 months in terms of adherence to ReMindCare, relapse prevention, hospital admissions, and visits to urgent care units compared with treatment as usual without the app. Duration: at least 1 month	ReMindCare: app that conducts daily evaluations of the health status of patients with early psychosis by offering quick questionnaires. Two types of questionnaires were included: daily questionnaires (3 daily questions assessing levels of anxiety, sadness, and irritability) and weekly questionnaires (18 weekly questions aimed at assessing adherence to medication, the presence of side effects from antipsychotic medication intake, the attitude toward medication intake, and the presence of prodromal psychosis symptoms)
Bucci et al. 2018 (37)	UK	36 participants (24 participants use Actissist plus treatment as usual and 12 participants used ClinTouch plus treatment as usual) Mean age of 20.21 years (SD = 7.37)—Actissist; Mean age of 18.33 years (SD = 7)—ClinTouch	Type of study: a single, blind, randomized controlled trial Aim: to test the safety, feasibility, and acceptability of Actissist and to provide preliminary evidence of intervention effects on clinical and functional outcomes Duration: 12 weeks	Actissist: a digital health intervention grounded in the cognitive model of psychosis that targets key early psychosis domains. Users can engage with spontaneously or in response to being prompted. It then collects responses from the user and wirelessly uploads user responses to a server. Actissist is divided in 2 parts, although presented as a single app. Firstly, at 3 pseudo-randomized time points per day, 6 days a week between 10.00 and 22.00, an auditory alert followed by a visual prompt is emitted from the app inviting participants to access the app. Part 2 includes a menu of multi-media options that act in a stand-alone fashion designed to complement and support the feedback from the intervention domains. This supplementary content contains information and activities including relaxation and mindfulness exercises, recovery stories (videos), a range of fact sheets (eg, low mood, anxiety, self-esteem), external links to web-related content (eg, TED talks), daily diary, and emergency contacts resources ClinTouch app is a symptom-monitoring app that triggers, collects, and wirelessly uploads symptom data to a server *Although the study addresses two apps, its focus is on Actissist, so only this one is described in more detailed
Eisner et al. 2019 (39)	UK	18 participants Mean age of 37.9 years (SD = 9.9)	Type of study: this study consisted of three phases. First, cross-sectional assessments characterized the sample and checked eligibility for the next phase. Second, eligible participants used ExPRESS for 6 months and received telephone calls from the researcher (prospective, longitudinal phase). Finally, after 6 months the acceptability of the study procedures was explored using qualitative interviews Aim: to investigate the feasibility and validity of adding “basic symptoms” to conventional early signs and monitoring these using a smartphone app Duration: 6 months	ExPRESS: an android smartphone app which prompts participants once a week to answer a personalized set of questions regarding psychotic symptoms, mood symptoms and early signs of relapse. The software was adapted from ClinTouch, a symptom-monitoring app assessing 12 Positive and Negative Syndrome Scale (PANSS) and 2 Calgary Depression Scale (CDS) items. They share their look, feel, and general functionality, but features such as item content and alert frequency differ
Fulford et al. 2020 (48)	USA	11 participants—focus groups; 8 participants—usability testing (including 3 participants from the focus groups) No data available about age mean and SD	Type of study: app development and pilot usability testing Aim: to develop a smartphone app designed to address social skill and motivation deficits in schizophrenia and test usability Duration: 14 days	Motivation and Skills Support (MASS): the app target social skills and social motivation in peoples’ day-to-day lives. It is tailored to 11 different social goals that fit into broad categories related to friend, family, and romantic relationships. These social goals were based on both traditional SST content and specific feedback from stakeholders. Video demonstrations of specific social skills that were relevant for these goals were also available through the app to support social goal completion. Within the app, participants were provided reminders of their social goal as well as information to help with goal planning. Specifically, each of the 11 goals were broken down into distinct steps that varied in difficulty
Garety et al. 2021 (43)	UK	361 participants (181—SlowMo app; 180—treatment as usual) Mean age of 43.1 years (SD = 11.7)—SlowMo app; Mean age of 42.2 years (SD = 11.6)—TAU; Mean age of 42.6 years (SD = 11.6)—Overall	Type of study: parallel-arm, assessor-blinded, randomized clinical trial Aim: to investigate the effects on paranoia and mechanisms of action of SlowMo app plus usual care compared with usal care only Duration: 12 weeks	SlowMo: a digitally supported CBTp consisting of 8 individual, face-to-face sessions (60–90 min) in accordance with a clinical manual. The intervention builds awareness of unhelpful fast thinking and supports individualized formulation. SlowMo then assists people with slowing down for a moment to find ways of feeling safer. Sessions are assisted by the SlowMo web app delivered using a touchscreen laptop, with interactive features including information, animated vignettes, games, and personalized thought bubbles. The web app synchronizes to a native android mobile app providing access in daily life to SlowMo strategies and individualized safer-thought bubbles
Gire et al. 2021 (44)	UK	12 participants Mean age of 24.83 years (SD = 4.83)	Type of study: mixed methods feasibility study Aim: to examine the acceptability and feasibility of a mobile phone application-based intervention for individuals with psychosis Duration: 6 weeks	TechCare App: system that assesses participants’ symptoms and responses in real-time and provides a personalised-guided self-help-based psychological intervention based on the principles of Cognitive Behaviorual Therapy (CBT), which could include participants’ preferred multimedia such as music, images, or video. The app used Experiential Sampling Methodology (ESM) as a research methodology, which allowed participants to record, subjective experiences in real-time of their thoughts, moods, and experiences of distress. The ESM research methodology was coupled with intelligent Real-Time Therapy (iRTT) uses the data gathered by ESM on a participants’ subjective experiences of distress and in response provides interventions to be delivered in real-time
Hanssen et al. 2020 (30)	The Netherlands	64 participants enrolled, 14 dropped out of the study for a variety of reasons, being in the study 50 participants (27 participants receiving ESM-derived personalized feedback and 23 participants without feedback) Mean age of 37.9 years (SD = 8.6)—feedback; Mean age of 40.3 years (SD = 10.9)—no feedback	Type of study: pre and posttest. Participants were randomly assigned to either of two groups: (1) one where the SMARTapp provided feedback according to the participants’ daily ESM entries, or (2) one where the SMARTapp included only ESM questionnaires without personalized feedback Aim: to examine the feasibility and effectiveness of an interactive smartphone application (with or without feedback) that aimed to improve daily-life social functioning and symptoms in schizophrenia spectrum disorders Duration: 21 days	SMARTapp (Schizophrenia Mobile Assessment and RealTime feedback application): this app was made using custom questionnaires which were built on the PsyMate™ platform (www.psymate.eu), which is a platform including a smartphone app, a cloud-based data storage and a reporting module, that allows customized collection of ESM data (thoughts, feelings, and behavior) in everyday life. The SMARTapp was identical for both groups, except that one group received personalized interactive ESM-derived feedback from the application in the form of two tailored prompts a day. The prompts provided suggestions for a certain activity or behavior change, depending on the previous ESM answers. The application provided feedback in the following categories: (a) psychotic symptoms, (b) social engagement, (c) health behavior (i.e., sleep, eating), (d) physical activity, and (e) mood and emotion. Feedback-prompts were programmed in such a way that even if ESM questions were answered in a similar fashion, participants did not receive the same prompt twice in a day. In the evening questionnaire, the feedback group was asked whether they acted upon the suggestions or not
Kidd et al. 2019 (35)	Canada	38 participants Mean age of 31.42 years (SD = 8.6)	Type of study: pre-post design and feasibility study of a schizophrenia-focused mobile application (medication adherence, personal recovery, and psychiatric symptomatology) Aim: to investigate feasibility and outcomes of a multifunction mobile app Duration: 1 month	App4Independence: a multi-feature app that uses feed, scheduling, and text-based functions co-designed with service users to enhance illness self-management. A4i functionality includes (i) addressing social isolation through personalized prompts, scheduling of activities, and connections to a range of resources relevant to social engagement. (ii) fostering engagement in the recovery process through evidence-informed content that makes suggestions and provides resources relevant to coping with psychosis symptoms, negative symptoms of schizophrenia, cognitive challenges, motivation, and anxiety as relevant to the individual. The content concentrations are determined by the above-mentioned algorithm. (iii) A peer-peer engagement platform that facilitates strategy/tip-sharing between A4i users (anonymous and moderated). (iv) Daily wellness and goal attainment check-ins to inform content delivery and highlight mental health trajectories. (v) Passively collected data on phone use as a proxy for sleep and activity levels
Kreyenbuhl et al. 2019 (51)	USA	7 participants Mean age of 47.6 years (SD = 10.4)	Type of study: short-term open trial Aim: to develop and assess preliminary acceptability and feasibility of a smartphone intervention to improve adherence to antipsychotic medication Duration: 2 weeks	MedActive: a smartphone application that reminds individuals to take their antipsychotic medications, stores and tracks information about their self-reported medication adherence, positive psychotic symptoms, and antipsychotic side effects, and allows them to research topics regarding schizophrenia and antipsychotic medications. It facilitates the active involvement of individuals with schizophrenia in managing their antipsychotic medication regimen by providing automated reminders for medication administration and tailored motivational feedback to encourage adherence, and by displaying user-friendly results of daily ecological momentary assessments (EMAs) of medication adherence, positive psychotic symptoms, and medication side effects for individuals and their psychiatrists. MedActive also consists of a clinician interface which is a secure website that enables psychiatrists to review their patients’ daily antipsychotic medication, positive psychotic symptoms, and antipsychotic side effects at any time
Krzystanek et al. 2019 (47)	Poland	290 participants (later 88 participants withdraw the study; (199 participants receiving smartphone with MONEO platform installed and 91 participants receiving the platform with functionality limited to monthly teleconsultation and performing cognitive training every 6 months) Mean age of 32 years (SD = 5.92)—MONEO; Mean age of 32.2 years (SD = 6.94)—MONEO with limited functionality	Type of study: multicenter, open-label randomized trial Aim: to test a smartphone-based MONEO platform designed to improve the clinical condition of paranoid schizophrenia patients Duration: 12 months	MONEO: a platform which consists of three modules: a web portal (for physician) available on a tablet or a computer, a smartphone application (for patient) available on a smartphone, and a web portal (for administrator), which sends automatic messages to the patient application—reminders about planned outpatient visits, the need to take medications or to conduct cognitive training, and information about received educational videos
Lewis et al. 2020 (38)	UK	81 participants (40 participants receiving ClinTouch and 41 participants receiving standard care) Mean age of 33.7 years—ClinTouch; Mean age of 35.3 years—standard care (SD = not reported)	Type of study: open randomized controlled trial Aim: to assess (i) acceptability of continuous monitoring to SMI patients and health professionals over 3 months; (ii) impact of active self-monitoring on positive psychotic symptoms assessed at 6 and 12 weeks; and (iii) the feasibility of detecting early warning signs of relapse Duration: 12 weeks	ClinTouch: smartphone-based platform to help persons with severe mental illness to manage their symptoms and prevent relapse. By asking users to rate their experience several times a day, ClinTouch provides real-time illness management solutions. Users can draw connections between triggers and symptoms, as well as get assistance in remembering to take medications. ClinTouch also tracks users over time so that early signs of relapse can be recognized sooner, enabling them to seek additional support and intervention
Lim et al. 2020 (52)	Australia	10 participants (2 dropouts) Mean age of 20.5 years (SD = 2.65)	Type of study: uncontrolled single-group design with three time points Aim: to develop a pilot digital smartphone application to target loneliness that is feasible and acceptable to young people with early psychosis Duration: 6 weeks	+Connect: a digital smartphone application which delivers positive psychology content daily. The aim of this content was to assist individuals to identify and harness their personal strengths, and to learn and practice positive interpersonal skills that could strengthen their current relationships. Themes included eliciting positive emotions, as well as showing kindness and reciprocity within relationship and social fears, which addresses social anxiety. When the application is opened, participants see a home screen, and are asked to log their mood using a mood evaluation tracker. They then proceed to the tasks which were delivered in one of four ways: (1) *via* text and images (e.g., an Instagram format); (2) shared experience videos featuring young people with lived experiences; (3) expert videos featuring academics introducing core concepts; or (4) actor videos featuring semi-professional actors modelling a range of social behaviors
Moitra et al. 2021 (53)	USA	10 participants (7 completed all measures and 1 participant was hospitalized) Mean age of 44.4 years (SD = 13.9)	Type of study: open trial Aim: to examine the initial feasibility, acceptability, and possible effects of the newly developed mobile intervention for patients with psychosis post-hospitalization. Duration: 1 month	Mobile After-Care Support (MACS): mHealth intervention for patients with psychosis during the transition from inpatient to outpatient care. Using CBTp-based strategies, the app was designed to monitor patients’ treatment adherence and symptoms and to intervene by providing brief, just-in-time interventions to support treatment adherence and participants’ use of healthy coping skills to manage their illness
Moore et al. 2020 (45)	Australia	12 participants No data available about age mean and SD	Type of study: qualitative study (nested in a pilot randomized controlled trial comparing a four-session smartphone-supported coping intervention, versus treatment-as-usual for people who hear persisting and distressing voices) Aim: to explore participants’ experiences of the smartphone-supported coping intervention, and to understand the impact of blending digital technology with a face-to-face intervention for distressing psychotic experiences from a user's perspective Duration: 8 weeks	movisensXS: app used to deliver ecological momentary assessment surveys and deliver ecological momentary intervention prompts. The intervention involved four semi-manualized face-to-face cognitive-behavioral therapy sessions with smartphone application monitoring and support between sessions. The app was used to deliver prompts to facilitate both self-monitoring and self-management of voice hearing experiences. The therapists and participants could collaboratively develop personalized coping strategies to embed into the application as prompts or reminders and then used in the deliver ecological momentary intervention phase of the intervention
Niendam et al. 2018 (40)	USA	76 participants Mean age of 18.8 years (SD = 3.7)	Type of study: longitudinal feasibility and validity study Aim: to determine feasibility and acceptability of implementing a smartphone application as an add-on tool in EP care and to evaluate the validity of self-report symptom data collected *via* a smartphone application in early psychosis Duration: up to 14 months	Ginger.io: a mHealth software comprising a smartphone application (“app”) and clinician Dashboard. The app can collect “active” data (i.e., self-report surveys sent to participants’ smartphones at designated times/days) and “passive” data (i.e., data gathered from participants’ smartphones without participant action) regarding phone calls made/received, SMS messages sent/received, and movement patterns (e.g., distance travelled in a day) based on Global Positioning System (GPS) data. Content of phone calls/SMS messages is never collected, and specific location data is never explicitly analyzed. The app served as a data collection tool only; participants could not contact their treatment team, or review their data *via* the app. Instead, active, and passive data for each participant were summarized on the Dashboard, a secure web-portal where clinicians reviewed participants’ data, including daily and weekly survey responses, survey completion rates, and passive data summaries. Dashboard notifications appeared when participants did not complete surveys more than 3 days in a row, or when passive data were not being collected from a participants’ phone. Clinicians also received Dashboard “alerts” when participants’ responses were considered clinically significant
Palmier-Claus et al. 2013 (54)	UK	24 participants Mean age of 33.04 years (SD = 9.5)	Type of study: randomized repeated-measure cross-over design study Aim: to explore patients’ understandings and perceptions of mobile phone based clinical assessment for psychosis, and how it might be implemented into their everyday lives and clinical care Duration: 6 days	No name: a designed software application for Android smartphones which displays self-report questions about symptomatology
Schlosser et al. 2018 (46)	USA	43 participants (22 participants receiving PRIME and 21 participants receiving treatment-as-usual/waitlist) Mean age of 24.32 years (SD = 2.6)—PRIME; Mean age of 23.79 (SD = 4.5)—treatment as usual/waitlist	Type of study: randomized controlled trial Aim: to test the efficacy of a new mobile intervention called PRIME (personalized real-time intervention for motivational enhancement) Duration: 12 weeks	PRIME: a mobile app intervention that includes a peer community, goal and achievement tracking, and cognitive behavioral therapy (CBT) based coaching. The intervention was designed to target the motivational system by utilizing social reinforcement to engage and sustain goal-directed behavior. The targeting of motivated behavior was hypothesized to require successful engagement of the various component process of reward processing, known to be disrupted in psychosis spectrum disorders. Participants worked towards self-identified goals with the support of a virtual community of age-matched peers with schizophrenia-spectrum disorders as well as motivational coaches
Simões de Almeida et al. 2019 (32)	Portugal	9 participants Mean age of 38 years (SD = 9.701)	Type of study: a pilot study Aim: to describe the development of weCope, a Portuguese mobile app for people with schizophrenia, and to present some preliminary data Duration: 8 weeks	weCope: an app that targets coping with voices, problem solving, goals setting and stress management. The symptom monitoring module share one of the goals of CBT including modifying patients’ distorted beliefs about delusions and hallucinations. Thus, the main objective is to monitor the frequency, intensity and duration of psychotic symptoms, their triggering events and the conditions that maintain them. The problem-solving module aims to identify and modify the maladaptive strategies to develop more helpful alternatives, and the user must identify the problem and describe it, presenting two possible solutions and selecting her/his preferred one. The anxiety management module purposes the reduction of factors such stress for a better control of the pathology and its symptoms—the user can explore different types of relaxation, such Jacobson Technique. In goal setting module, the user has to define the goal, deadlines, and the support that he will require, so he can have an attitude of empowerment in their recovery
Steare et al. 2020 (33)	UK	40 participants (20 participants used the app and 20 participants were the control group) Mean age of 29.4 years (SD = 9.7)—My Journey app; Mean age of 30 (SD = 10.1)—control group	Type of study: a two-arm unblinded feasibility randomized controlled trial Aim: to test the feasibility and acceptability of a randomized controlled trial to evaluate a Smartphone-based self-management tool in Early Intervention in Psychosis services Duration: from 4 to 12 months (median 38.1 weeks)	My Journey 3: a smartphone app developed for adults accessing Early Intervention in Psychosis (EIP) services. The aim of the intervention is to develop users’ self-management skills to help them to achieve self-determined recovery goals and avoid future relapses. My Journey 3 is suitable for independent use, but also designed to be used with support from EIP service clinicians who will be able to assist with the completion of the self-management components and initial set-up. It is the developers’ aspiration for My Journey 3 to be used initially in collaboration with EIP service clinicians, and for it to support continuing self-management after users have been discharged from EIP services
Steare et al. 2021 (34)	UK	34 participants (21 patients and 13 clinicians) Mean age of 29.8 years—patients (SD = not reported) Clinicians’ data not reported	Type of study: qualitative study—semi-structured one-to-one interviews as part of a feasibility trial Aim: to explore the acceptability of My Journey 3 through interviews with both clinical providers and service users Duration: from 4 to 12 months (median 38.1 weeks)	My Journey 3: an app to increase the implementation of supported self-management for adults with first-episode psychosis. Also, Users of the app can create and store a relapse prevention plan with the aid of an early intervention psychosis service clinician. My Journey 3 features a symptom tracker, information on mental health, medication, and mental health services in a psychoeducation section of the app including short videos. Medication adherence can be tracked *via* a “Pill Tracker”
Taylor et al. 2022 (50)	UK	12 participants (3 dropout) Mean age of 35.57 years (SD = 10.88)	Type of study: a feasibility and acceptability study Aim: to evaluate the feasibility, acceptability and potential usefulness of a guided, smartphone-based CBT intervention targeting sleep disturbance for individuals with psychosis Duration: 6 weeks	ExpiWell: the intervention consisted of a system-initiated, pre-scheduled programme (“My Sleep Programme”) and a user-initiated, access-any-time “Resources Section”. My Sleep Programme consisted of six core weekly modules, and one further participant-chosen module (“Managing Worry” or “Coping with Voices”). Each required up to 30 min to complete. New content was delivered in the form of “surveys”, to which participants were alerted with a home screen notification. The intervention was interactive and conversational; utilizing a range of question-response styles to actively involve the user, with supportive feedback provided. Content was tailored to participant responses using branching logic. This format has similarly been used in other app-based interventions designed for individuals with psychosis. The Resources Section enabled participants to re-access module summaries, and access audio-guided relaxation and mindfulness exercises
Terp et al. 2018 (42)	Denmark	27 participants (only 13 participated in this study evaluation) Mean age of 24.8 years (SD = not reported)	Type of study: qualitative (constituted the third phase of a participatory design process) Aim: to explore how young adults recently diagnosed with schizophrenia used and perceived a smartphone app as a tool to foster power in the everyday management of living with their illness Duration: 1 year	MindFrame: a smartphone app that allows young adults diagnosed with schizophrenia to access resources to aid their self-management. Every day, individuals are required to fill out a self-assessment on their smartphone; these assessments will reveal the state of the individual's mental health. The data collected on the self-assessment will be displayed on the smartphone app as a graph over the course of 14 days. The clinician can also access the patient's self-assessments through the clinical web portal enabling him/her to identify any patterns and make possible connections between certain behaviors and the symptoms they may trigger. With the data from the self-assessments, the system can also identify triggers and early warning signs notifying both, the patient, and the clinician

### Strategies to improve user engagement

Not all the studies included in the scoping review focused engagement concretely, but all of them address this issue in some way (Table 2). As all this data is the result of studies carried out, downloading the apps is something already integrated into the process. What is important to comprehend is how to enhance all the ways users interact with the apps, from buttons they click to time spent with the app open.

**Table 2:** User engagement indicators.

Authors/Year	User engagement strategies	Factors	Measures	Main findings
Achtyes et al. (26)	Notifications/prompts	Age Gender Education level Technical support	Demographic variables were assessed for each participant Data about app usage (each time a participant accessed FOCUS, the date and time-stamped login was recorded within the tool)	Subjects in this study accessed FOCUS half the time (38,139 times, 50.6%) during off-hours, indicating that approximately half the “dose” of this intervention occurred when the clinic was closed, an important extension of care during times when access to a clinician may be difficult or impossible A subset of “high utilizers” (*n* = 152, 43.8%) self-initiated use of all five FOCUS modules both on- and off-hours “High utilizers” tended to be women, >35 years old, and had a high school diploma or greater Patients older than 35 years engaged well with FOCUS when given ongoing technical support from the mental health technology coach
Ben-Zeev et al. (27)	Notifications/prompts	30-min training session (demonstration focused on in-the-moment use and selection of resources from the different on-demand options. Users then had the opportunity to practice using FOCUS and ask questions as needed)	Demographic and clinical variables were assessed for each participant [Positive and Negative Syndrome Scale (PANSS) for Schizophrenia; Beck Depression Inventory, Insomnia Severity Index, Brief Assessment of Cognition in Schizophrenia, Brief Medication Questionnaire] 26-item self-report acceptability/usability measure comprised of adapted items from the System Usability Scale Post Study System Usability Questionnaire Technology Assessment Model Measurement Scales Usefulness, Satisfaction, and Ease questionnaire Participants were asked to rate their agreement with a series of statements about the intervention	Completers used the system on 86.5% of days they had the device, an average of 5.2 times a day Approximately 62% of use of the FOCUS intervention was initiated by the participants, and 38% of use was in response to automated prompts Approximately 90% of participants rated the intervention as highly acceptable and usable
Ben-Zeev et al. (36)	Notifications/prompts	Study staff call participants if it appears that there is a problem with passive data collection	Demographic variables and comprehensive baseline assessment of symptoms and functioning (semi-structured clinical interviews and questionnaires) App data (active and passive user assessments)	Participants had unique digital indicators of their psychotic relapse. For some, self-reports provided clear and potentially actionable description of symptom exacerbation prior to hospitalization. Others had behavioral sensing data trends (e.g., shifts in geolocation patterns, declines in physical activity) or device use patterns (e.g., increased nighttime app use, discontinuation of all smartphone use) that reflected the changes they experienced more effectively Not all participants in the ongoing CrossCheck study complete a full year of data collection, adhere to the study protocol (i.e., carry the smartphone with them, charge the battery, complete self-reports regularly), or experience hospitalizations
Ben-Zeev et al. (28)	Notifications/prompts	Brief weekly calls from an mHealth support specialist who assisted them in all technical and clinical aspects of the intervention	Demographic and clinical variables were assessed for each participant (Symptom Checklist, Beck Depression Inventory, Psychotic Symptom Rating Scales, Recovery Assessment Scale) Satisfaction 5-item questionnaire Data about app usage (engagement in treatment was calculated for each participant weekly by using his or her FOCUS use data; participants were considered engaged if they used the app on at least five of 7 days a week (that is, approximately 70%). A FOCUS “use” event is recorded as such only if, following a prompt, participants elect to engage in a clinical status assessment or if they self-initiate one of the FOCUS on-demand tools)	Averaging across all participants assigned to FOCUS, participants used the app on 5.4 ± 2.4 days in the first week of the intervention, on 4.6 ± 2.7 days in the third week, on 4.3 ± 2.7 days in the sixth week, on 3.9 ± 2.7 days in the ninth week, and on 3.8 ± 2.9 days in the last week Satisfaction was high
Ben-Zeev et al. (29)	Notifications/prompts Use of videos	Concerns about privacy Apprehensive about using video interventions in public for fear of public stigmatization	Demographic and clinical variables were assessed for each participant (Psychotic Symptom Rating Scales, Beck Depression Inventory) Semi-structured interview about their experiences with the video and written interventions 11-item measure in which they were asked to indicate their intervention modality preference 12-item measure examining usability, acceptability, and satisfaction	On average, participants found videos to be more personal, engaging, and helpful in supporting their illness management. Participants responded to 67% of system-delivered prompts to engage FOCUS-AV, and 52% of FOCUS-AV use was initiated by the users. On average, participants used interventions 6 days a week, four times daily. Participants used video functions an average of 28 times. They chose video over written interventions on 67% of the times they used on-demand functions but opted for written content 78% of the times they responded to pre-scheduled prompts Written interventions were rated as more favorable in letting users proceed at their own pace
Bonet et al. (41)	Notifications/prompts Clinicians contact patients by phone in response to preset alarms	Clinicians’ workload Limited efforts to integrate these apps into care settings Excessive eHealth communications could be regarded as intrusive or irritating	Demographic and clinical variables were assessed for each participant (Clinical Global Impression Severity of Illness scale, Global Assessment of Functioning, Positive and Negative Syndrome Scale (PANSS), Premorbid Adjustment Scale) Feasibility, efficacy, compliance, and engagement measures: number of times patients answered the questionnaires when presented and number of months using the app, patients’ dropouts, plus number of urgent consultation requests	Only 20% (12/59) of patients from the ReMindCare group had a relapse, while 58% (18/31) of the TAU patients had one or more relapses (*χ*^2^ = 13.7, *P *= .001) Moreover, ReMindCare patients had fewer visits to urgent care units (*χ*^2^ = 7.4, *P *= .006) and fewer hospitalizations than TAU patients (*χ*^2^ = 4.6, *P *= .03). The mean of days using the app was 352.2 (SD 191.2; min/max: 18–594), and the mean of engagement was 84.5 (SD 16.04)
Bucci et al. (37)	Notifications/prompts Participants could view written and visual “in-app” instructions Multiple messages and images associated with each exchange minimize boredom and repetition within the app Users can customize the aesthetics of the Actissist interface	45-min phone set-up training session All participants received a weekly phone call from the project manager to troubleshoot equipment functions	The primary outcome was feasibility, which was assessed in terms of uptake (the proportion of eligible participants consenting to the study), attrition, the proportion of participants completing user, and alert-initiated data entries across participants (>33% data points), and the proportion continuing for 12 weeks (both arms) Satisfaction with technology was also measured Acceptability of the Actissist intervention was assessed *via* participant feedback Other secondary outcome measures related to functioning and symptom were assessed	Actissist was feasible (75% participants used Actissist at least once/day; uptake was high, 97% participants remained in the trial; high follow-up rates), acceptable (90% participants recommend Actissist), and safe (0 serious adverse events), with high levels of user satisfaction. Treatment effects were large on negative symptoms, general psychotic symptoms and mood. The addition of Actissist conferred benefit at post-treatment assessment over routine symptom-monitoring and treatment as usual
Eisner et al. (39)	Notifications/prompts (above-threshold increases in app-reported psychotic symptoms prompted a telephone interview to assess relapse)	Participants were telephoned by the researcher (weekly for 4 weeks; monthly thereafter) to encourage participation and troubleshoot any difficulties with app use	Demographic and clinical variables were assessed for each participant (Schizophrenia Proneness Instrument Adult Version interview, Fear of Recurrence Scale, Hospital Anxiety and Depression Scale, Psychotic Symptom Rating Scales) Qualitative interviews App engagement was measure through percentage of assessments completed	Participants completed 65% app assessments and 58% telephone interviews. App items showed high concurrent validity with researcher-rated psychotic symptoms and basic symptoms over 6 months. There was excellent agreement between telephone call and face-to-face assessed psychotic symptoms. The primary relapse definition, based on telephone assessment and case notes, compared well with a case note-only definition but had better specificity. Mixed-effects models provided preliminary evidence of concurrent and predictive validity: early signs and basic symptoms were associated with most app-assessed psychotic symptom variables the same week and with psychotic symptoms variables 3 weeks later; adding basic symptoms to early signs improved model fit in most of these cases
Fulford et al. (48)	Goal setting Push notifications administered three times per day (morning, mid-day, and evening) directed users to app content focused on their identified social goal and steps Provide more specific structure/guidance on steps to achieve social goals, and increasing in-person check-ins during app use Use of videos	Support for technical problems with the smartphone/app	Clinician interviews Focus groups with patients Usability test: quantitative and qualitative feedback to inform subsequent revision of the app through an online survey, regarding their experience with the app, including discussing barriers and facilitators to using the app and any additional feedback to be implemented in a future iteration of the app	Preliminary evidence of acceptability and feasibility of the MASS app is promising given the limited available mobile apps focused on addressing social skills and motivation specifically through a standalone app Key features that were identified by participants as helpful included the video content, the reminders of steps necessary to complete their goal, and the ease of using the app. Some notable suggestions for improving the app experience included decreasing the number of daily notifications, adding more video content, providing more specific structure/guidance on social goals, and increasing in-person check-ins during app use
Garety et al. (43)	Use interactive features including information, animated vignettes, games, and personalized thought bubbles Notifications/prompts	Inclusive, human-centered design	Demographic and clinical variables were assessed for each participant (Paranoid Thoughts Questionnaire, Psychotic Symptom Rating Scales, Maudsley Assessment of Delusions Schedule, Fast and Slow Thinking Questionnaire, Warwick-Edinburgh Mental Well-being Scale, Manchester Short Assessment of Quality of Life, Brief Core Schema Scales, Penn State Worry Questionnaire) Semi structured interviews investigated the feasibility and potential acceptability of the intervention Mobile app adherence was operationalized as at least 1 home screen interaction after a minimum of 3 therapy sessions and was recorded by system analytics	The mean (SD) number of SlowMo sessions attended was 6.8 (2.6), increasing to 7.3 (1.9) for those attending 1 or more sessions. Among the 181 participants in the SlowMo arm, 145 (80.1%) completed all 8 therapy sessions, 13 (7.2%) attended no sessions, and 23 (12.7%) discontinued therapy between sessions 1 and 7. Mean (SD) session duration, including behavioral work, was 75 (29) minutes. SlowMo plus TAU was not associated with greater reductions than TAU alone in the primary outcome of GPTS total paranoia score at 24 weeks (Cohen *d*, 0.20; 95% CI, −0.02 to 0.40; *P* = .06)
Gire et al. (44)	Notifications/prompts Use of multimedia such as music, images, or videos If the App detected low mood/paranoia, participants were offered tailored interventions; when symptoms are exacerbated causing severe distress, the crisis response may include contacting the early intervention services or an agreed designated contact	Financial implications of mobile ownership and connectivity to the internet Confidentiality and security	Demographic and clinical variables were assessed for each participant (Positive and Negative Syndrome Scale (PANSS), Psychotic Symptom Rating Scales) The success criterion for feasibility was the recruitment of ≥50% of eligible participants the acceptability of the intervention was assessed based on the amount of engagement and usage of the TechCare App, with the success criterion for compliance being set at ≥33%	A total of 83.33% (*n* = 10) of participants completed the 6-week feasibility study, with 70% of completers achieving the set compliance threshold of ≥33% engagement with the TechCare App system. Analysis of the qualitative data suggested that participants held the view that the TechCare was both an acceptable and feasible means of delivering interventions in real-time
Hanssen et al. (30)	The application was personalized for all participants, both with and without feedback, according to the personal preferences of the participant—they filled in enjoyable activities, several social contacts, comforting thoughts, and relaxing activities Notifications/prompts	Some participants indicated that there were too many beeps during the day and that they sometimes felt disturbed in their activities by the beep On day 2 and day 7 participants were contacted by phone to check for technical difficulties and whether they had any additional questions. A contact number was provided for technical support	Demographic and clinical variables were assessed for each participant (Positive and Negative Syndrome Scale (PANSS), Community Assessment of Psychic-Experiences, Social Functioning Scale, and two subtests of the Wechsler Adult Intelligence Scale) Six short Experience Sampling Method (ESM) questionnaires daily when prompted by a beep, for a duration of 3 weeks and an evening questionnaire Evaluation questionnaire of the app	The response rate was 64% for the ESM questionnaires. In the feedback group, participants indicated that on 49% of the ESM days they acted on at least one personalized feedback prompt per day. Momentary psychotic symptoms significantly decreased over time only in the feedback group. Momentary loneliness and questionnaire-assessed psychotic symptoms decreased over time, irrespective of feedback. Participants rated the SMARTapp as easy to use (94%) and appealing (95%), indicated that questions were clear (80%), and generally felt that they could reflect their experiences well through the questions provided by the application (68%). Seventy-four percent of the participants said they used the coping tips, and 54% found them useful (43% neutral, 3% not useful)
Kidd et al. (35)	Notifications/prompts Content customization regarding daily wellness and goal attainment check-ins to inform content delivery and highlight mental health trajectories Medication, appointments, and event reminders are custom set by the user during onboarding and can be added throughout app use A peer-peer engagement platform that facilitates strategy/tip-sharing between A4i users (anonymous and moderated) Passive collect data on phone use as a proxy for sleep and activity levels	Illness severity Gender Age	Demographic and clinical variables were assessed for each participant (Brief Symptom Inventory, Personal Recovery Outcome Measure, Brief Adherence Rating Scale) The mHealth use and utility scale used by Ben-Zeev and colleagues was employed with minor modification to specify A4i use Qualitative data included the use of field notes to capture information gathered in weekly check-in contacts and, at post assessment, a semi-structured interview was used to capture what was more and less helpful/engaging in participants’ experiences of using A4i Metrics collected through the app included overall time used, time and frequency of using specific functions, frequency of app refresh, a sleep-proxy metric, and daily wellness check-in responses. Descriptive data analysis included a descriptive profile of participants, their app use, and ratings of app utility	Among the 38 individuals with a primary psychosis who participated, there was no research attrition and classic retention on the app was 52.5%. Significant improvement was observed in some psychiatric symptom domains with small-medium effects. Significant change in recovery engagement and medication adherence were not observed after controlling for multiple comparisons. Those who interacted with the app more frequently were more depressed and had higher hostility and interpersonal sensitivity at baseline. Satisfaction with the app was high and qualitative feedback provided insights regarding feature enhancements
Kreyenbuhl et al. (51)	Notifications/prompts Personalized motivational feedback Use of gamification	Informed by the Information-Motivation-Behavioral (IMB) Skills Model and using the iterative process of user-centered design Possibility to integrate the interface information into a patient's medical records (more work is needed to understand how to better enable service systems and providers to integrate real-time patient information into existing workflows to facilitate measurement-based care)	During the 2-week study period, all patient-participant use of the application (i.e., button presses, responses EMAs) and psychiatrist-participant use of the clinician interface were continuously uploaded to a secure study server At follow-up, psychiatrist- and patient-participants completed surveys querying them about the acceptability and likeability of the application and clinician interface, respectively The primary outcomes of the open trial were the feasibility and acceptability of the application and clinician interface	MedActive was determined to be both feasible and acceptable, with patient participants responding to 80% of all scheduled EMAs and providing positive evaluations of their use of the application. Psychiatrist participants were interested in viewing the information provided on the MedActive clinician interface but cited practical barriers to regularly accessing it and integrating into their daily practice
Krzystanek et al. (47)	Notifications/prompts Software also contained a library of videos and audiobooks accessible to the patients at will	Patients were able to report the need for a televisit Age Illness severity Reduced cognitive functions	The clinical status was measured using the Positive and Negative Syndrome Scale (PANSS), Calgary Depression Scale for Schizophrenia, and Clinical Global Impression-Severity clinical scales The number of hospitalizations and visits to outpatient clinics was gathered directly from the MONEO platform Medical adherence was monitored based on feedback messages sent by the patient	Adherence data gathered for the 12-month study on the MONEO platform ranged from 61% to 85% Patients using the full version of the MONEO platform exhibited a pronounced reduction in the schizophrenic symptoms Use of the MONEO platform did not influence the rate of hospitalization and visits to outpatient clinics
Lewis et al. (38)	Notifications/prompts	Illness severity	Demographic and clinical variables were assessed for each participant (Positive and Negative Syndrome Scale (PANSS)) Qualitative analyses (interviews) assessed acceptability of the system	Participant's adherence rate (defined as responding to >33% of alerts) was 84% Of 38 participants who completed 12 weeks of the trial, three (8%) reported significant events: 1 reported increased anxiety prompted by questions; 1 reported increased irritation due to the alert beeps, and 1 had their charger explode Qualitative analyses supported the acceptability of the system to participants and staff App use was associated with psychotic symptom improvement in recent-onset participants, but not those with longstanding illness, supporting the notion of improved self-management
Lim et al. (52)	Use of gamification (e.g., points, challenges, badges) to encourage participant engagement Personalization Relatability of content In-app feedback functions Use of video Youth-friendly design and navigation	Age User participation in design Fits with routine	Demographic and clinical variables were assessed for each participant (Positive and Negative Syndrome Scale (PANSS), Calgary Depression Scale for Schizophrenia, Social Skills Performance Assessment, Revised UCLA Loneliness Scale, Social Interaction Anxiety Scale, Scales of Psychological Well-being) A series of questionnaires were created to assess acceptability, feasibility, and usability A 20-item questionnaire designed for the study was used to assess how helpful each module was for the participants	+Connect helped participants to increase their social confidence, enjoy life, look forward to being with other people, and feel more connected with others Participant interviews supported these results, with participants highlighting the app's strengths in providing useful information, stimulating self-reflection, fostering positive affect, and encouraging transfer of skills into their social interactions Participants completed 95.47% of the +Connect exceeding the *a priori* criteria of app completion (33 out of 42 days). Eight out of the ten participants also remained engaged in the program 3 months following the end of treatment assessment. Of the ten participants, two reported early difficulties integrating the use of the app into their routine Preliminary findings indicated that +Connect yielded high levels of acceptability and feasibility
Moitra et al. (31)	Notifications/prompts	Lack of technology fluency Access to Internet Technical support	Demographic and clinical variables were assessed for each participant (Antipsychotic Medication Beliefs and Attitudes Scale, Brief Adherence Rating Scale, Brief Coping Orientation to Problems Experienced, Brief Psychiatric Rating Scale, World Health Organization Disability Assessment Schedule 2.0) System usability scale, Client Satisfaction Questionnaire-8, and the Usefulness, Satisfaction and Ease of Use Questionnaire	Participants completed about one session per day on average as expected. Overall, measures of MACS usability and satisfaction were positive Participant feedback was mostly positive as many noted the benefits of being prompted to reflect on their symptoms and functioning, as well as in receiving brief CBTp-based support
Moore et al. (45)	Notifications/prompts Personalization	Unexpected technology issues Therapeutic relationship—app as an extension of the therapist	Semi-structured interview	Participants perceived Ecological Momentary Assessment and Intervention (EMA/I) technology as helping capture their experience more accurately and communicate this more effectively to the therapist, which, in combination with coping prompts developed in-session, deepened the therapeutic relationship
Niendam et al. (40)	Active and passive data collection Dashboard notifications appeared when participants did not complete surveys more than 3 days in a row, or when passive data were not being collected from a participants’ telephone	Monetary incentives (participants were paid $0.50 per daily and $1.50 per weekly survey, i.e., maximum $20 monthly payment) Device breakage/loss	Demographic and clinical variables were assessed for each participant (Brief Psychiatric Rating Scale) Participants completed self-report surveys at the end of the study evaluating satisfaction and perceived impact on clinical care	High survey completion rates (average 77% weekly; 69% daily) were not related to symptom severity or length of time in treatment at the clinic, indicating that even symptomatic individuals early on in treatment are able and willing to engage in smartphone-based surveys as part of their treatment 97% of participants reported that the app was easy to use 40% of participants indicated the app helped them remember to take their medication and increased motivation for symptom management and treatment engagement. This suggests that, for some individuals, simply tracking symptoms and medication adherence *via* an app may be an intervention in and of itself
Palmier-Claus et al. (55)	Notifications/prompts	Fits with routine Illness severity Repetitiveness of the questions Socioeconomic status Reading ability Researcher support (phone calls)	Demographic variables were assessed for each participant Qualitative interviews were conducted to explore participants’ perceptions and experiences of the devices, and thematic analysis was used to analyze the data. Three themes emerged from the data: (i) the appeal of usability and familiarity, (ii) acceptability, validity, and integration into domestic routines, and (iii) perceived impact on clinical care	Compliance as defined by completion of at least 33% of all possible data-points over 7 days was 82%. ClinTouch is a valid form of self-assessment, which could facilitate the real-time monitoring of symptoms in schizophrenia in research and clinical management settings. In addition to overcoming the constraints of rater training and limited reliability, recall bias and averaging, it potentially offers advantages over semi-structured interview administered scales allowing finer-grained analysis over briefer time periods, with potential inclusion of external contingency data, diurnal and short-term variability and adding in of other behavioral data gathered by the same device, such as sleep pattern and activity
Schlosser et al. (46)	Motivation coaches Use of gamification (challenges) Goal setting Peer-peer community (users may send messages directly to each other and can also capture and share positive, spontaneous moments in their daily life with the whole PRIME community short bio) Personalization	Technical support	Demographic and clinical variables were assessed for each participant (Positive and Negative Syndrome Scale (PANSS), Trust Task, Motivation and Pleasure Self-Report Scale, Role Functioning Scale, Quality of Life Scale—Abbreviated, Dysfunctional Attitudes Scale, Beck Depression Inventory, Revised Self-Efficacy Scale) PRIME acceptability was assessed during an exit interview at the 12-week time point (post-trial) where participants rated their satisfaction with the specific features of PRIME. To evaluate feasibility, the following metrics were used: login frequency (average number of days logged in per week), average number of challenges completed (both overall and by individual challenge category), challenge completion percentage, and the average number of peer and coach interactions. To determine PRIME acceptability, the average ratings from the PRIME satisfaction survey were assessed. To investigate PRIME feasibility, descriptive statistics for the following PRIME metrics were examined: login frequency, challenges completed, spontaneous and goal achievement moments, peer and coach interactions, and active use rate	The overall 74% retention rate for the treatment (and 88% retention post-intervention), demonstrated that this intervention was very well tolerated. Participants rated their overall satisfaction with PRIME highly. On average, participants logged in a little over 4 day/week. Over a 12-week period, participants were highly engaged in the platform, with 5,152 direct messages sent from participants to coaches. In terms of peer-to-peer interactions, participants-initiated interactions with each other a total of 497 times. Participants initiated about 10 interactions with coaches for every initiated peer interaction. All 38 participants initiated at least 1 message to a coach and 13 (33%) initiated more than the average of 128.8 coach interactions. Participants completed an average of 1.5 challenges per week
Simões de Almeida et al. (32)	Goal setting Contact with therapists in crisis situations	Age Data security Onset of illness Context of care delivery (receiving ambulatory care) Illness severity Influence of clinicians User participation in design	Demographic and clinical variables were assessed for each participant (Positive and Negative Syndrome Scale (PANSS), Recovery Assessment Scale, Empowerment Scale, General Self-Efficacy Scale, Social Support Satisfaction Scale, Personal and Social Performance Scale) A usability brief questionnaire was also applied, asking participants their ability to use de app, satisfaction, and difficulties	Concerning the usability, most participants (45%) used the app two or three times a week and recognize the “Anxiety Management” and “Goal Setting” modules and the possibility to exchange messages with the therapist the modules being most used by them. Participants showed that were satisfied with the weCope system, considering the application useful for the management of the disease (89%), and 78% said they would continue to use the app
Steare et al. (33)	Goal setting Notifications/prompts	Age Regular smartphone use patterns Technical difficulties Influence of clinicians (clinicians were asked to discuss recovery goals and relapse prevention plans in routine appointments with participants, and assist with entering these into the appropriate My Journey 3 sections)	Demographic and clinical variables were assessed for each participant (Positive and Negative Syndrome Scale (PANSS), Social Outcomes Questionnaire, Mental Health Confidence Scale, Questionnaire about the Process of Recovery, Warwick-Edinburgh Mental Well-being Scale) DIALOG scale, Service Engagement Scale, app patient records and assessments through the app Semi-structured interview	83% and 75% of participants were retained in the trial at the 4- and 12-month assessments. All treatment group participants had access to My Journey 3 during the trial, but technical difficulties caused delays in ensuring timely access to the intervention. The median number of My Journey 3 uses was 16.5 (IQR 8.5–23) and median total minutes spent using My Journey 3 was 26.8 (IQR 18.3–57.3)
Steare et al. (34)	Communication with peers and clinicians Notifications/prompts	Age Data security and privacy Recovery stage Illness severity	Demographic variables were assessed for each participant DIALOG scale, Service Engagement Scale Clinical semi-structured interviews assess feasibility and acceptability of the intervention and barriers and facilitators to its use To assess acceptability of the intervention and user engagement, My Journey 3 usage data were collected for all participants in the treatment group from the training session until the 12-month time point	Many service user participants found My Journey 3 to be acceptable. The symptom and medication trackers were described as helpful. A smaller number of service users disliked the intervention. Clinicians tended to report that My Journey 3 was a potentially positive addition to service users’ care, but they often felt unable to provide support due to competing demands in their work, which in turn may have impacted acceptability and usage of the app
Taylor et al. (50)	Goal setting Supportive feedback Content was tailored to participant responses using branching logic Notifications/prompts	30 min therapist support weekly (included “technical”, “use” and “clinical” support to overcome any barriers related to engagement, understanding and implementation of strategies as required) Ease of remembering to use Fits with routine	Demographic and clinical variables were assessed for each participant (Pittsburgh Sleep Quality Index, Paranoid Thoughts Scale, Specific Psychotic Experiences Questionnaire, Depression, Anxiety and Stress Scale 21-itens, Warwick-Edinburg Mental Well-being Scale, Work and Social Adjustment Scale) Acceptability of the intervention was assessed by app engagement, an Experience Feedback Questionnaire and a semi-structured interview focused on the participant's experience of the app, barriers to engagement and their perception of the impact of the intervention	It is feasible and acceptable to deliver therapist-guided CBT for sleep problems by smartphone app for individuals with psychosis. On average, each participant engaged with 5.6 of 7 available modules. Engagement declined over time: all participants (*n* = 13; 100%) engaged with the week one modules, reducing to 61.5% (*n* = 8) by week 6. Responses to reminders similarly declined over time, starting at a mean of 44.6% (SD = 33.8%) completed in week 1, reducing to 29.5% (SD = 36.7%) at week 6. On average, participants engaged with 13.7 (34.3%; SD = 10.4) of the 40 reminders. Qualitative feedback indicated the intervention was considered helpful and would be recommended to others
Terp et al. (42)	Personalization Notifications/prompts	Illness severity User participation in design Fears and worries of restraint (surveillance/data sharing)	Semi-structured interviews	The findings demonstrate that young adults diagnosed with schizophrenia are amenable to use a smartphone app to monitor their health, manage their medication, and stay alert of the early signs of illness exacerbation. This may empower them to stay on track with their illness, thus in control of it. Participants in the evaluation described MindFrame as easy and intuitive to use. Some participants terminated use within 1 month (*n* = 5), others terminated use within 2–3 months (*n* = 4), and others used MindFrame for 6–12 months, terminating their use when the intervention period stopped (*n* = 4). Reasons given for self-initiated termination of MindFrame included boredom, lack of motivation and energy, fatigue, and problems quantifying their mental health

Almost every app uses push notifications or prompts, except the one from Simões de Almeida et al. (32), and the frequency of notifications are usually at least daily. Personalization and customization were also a strategy used in ten studies (30, 35, 37, 42, 45, 46, 48, 50–52) and could be associated to the design or to the content as well. Another common strategy presented in six studies is goal setting, i.e., present a goal-oriented process (32, 33, 35, 46, 48, 50). Some studies relied on game-like features (e.g., points, challenges, badges) (43, 46, 51, 52) or the use of different multimedia formats (e.g., videos, music, images) (29, 37, 43, 44, 47, 48, 52) to encourage participant engagement. Benefits to long-term engagement may occur using real-time data monitoring and passive monitoring. Peer-to-peer communication (34, 35, 46), access to professionals (32, 34, 41), reliability of content (52), and the quality of feedback received (50, 52), were also important strategies used to improve user engagement in these included studies.

### Factors that influence user engagement

There are three factors that may influence user engagement that constantly arise from these studies: age, illness severity, and technical support. Other demographic variables may can play a role in engagement namely gender (26, 35), education level (26), and socioeconomic status (55). Issues about data privacy and security and integration with other medical record systems were also described (29, 32, 34, 42, 44, 51). There are also some putative factors which could influence engagement as well, such as whether patients were involved in the app design process, incentives for participation, etc. (32, 40, 42, 43, 52). Most of these apps included at some stage user involvement in the development process and in most of the studies, participants were not monetarily incentivized to engage in interventions but were compensated for completing assessments. Any engagement strategies used should consider the need for initial training for using the app (including training sessions, smooth onboarding, possibility to ask questions and contact a support team to manage any technical difficulties) since some users could lack digital literacy/fluency (26–28, 30, 31, 33, 36, 39, 46, 48, 55). The fact that the use of the app fits well into the user's routine and that this use is encouraged by the clinician (or based on theoretical foundations and evidence) are also factors that influence engagement (32, 33, 50, 52, 55). Moreover, it is important to consider that user motivation to use mHealth may decline over time.

### Measures for user engagement

Included studies evaluate user engagement indicators with different methodologies, from the criteria (such as subjective ratings and objective data) to the means of assessment (such as a survey, interview, or usage data). Besides metrics collected through the app (including overall time used, time and frequency of using specific functions, frequency of app refresh, daily check-in responses, …) (26, 28, 30, 32, 35–37, 39, 41, 43, 44, 46–48, 51), most of studies used questionnaires and semi-structured interviews created to assess satisfaction, acceptability, feasibility, and usability (27, 29, 30, 32–35, 37, 39–43, 48, 50, 55). Some studies used validated instruments such as System Usability Scale (27, 31), Usefulness, Satisfaction and Ease Questionnaire (27, 31) or Service Engagement Scale (33, 34).

## Discussion

The findings of this scoping review have several implications for future research and give indications for designing and implementing mobile mental health applications.

First, it is important to note that in this context, engagement is measured by the frequency the user interacts with the app and by the impact that the use of the app has on the user's health (17).

Some human–computer interaction researchers define user engagement as catching and keeping the attention and interest of technology users and the cognitive, emotional, and temporal investment made by users (10), which should be adapted to this field, since people with schizophrenia have fluctuating needs. In this perspective, disengagement may be the visible result of a user that has fulfilled their goals using a health app, which should be seen as a natural part of the usage cycle. However, there may be the need to induce continued engagement to avoid relapse or maintain a given stage of evolution, which suggests that the app usage frequency pattern is as important as how the app is used by the user (52). Some apps might be designed for people to use them every day, whereas others might be designed for more emergent yet infrequent situations. O’Brien and Toms (56) in their conceptual framework of engagement with technology, drew attention to the cycles of engagement, disengagement, and reengagement that persists over time. The optimal “dose” of engagement is still unclear in the field of digital mental health interventions (53) and the time spent on a digital tool varies between different types of interventions, person, or context (57). Moreover, lack of engagement could be a good sign if it means that the users have achieved their recovery goals, or that the users are utilizing other resources that may be more helpful in each moment or that the app helped empower the users to seek traditional forms of therapy.

Many strategies are used to increase engagement, but the most common is the use of notifications that are a ubiquitous feature of mHealth apps, reminding the user to complete some action, informing about some event, or asking for information, which by itself increases the chances of data collection (58). However, the characteristics of message prompts have a huge impact on the effectiveness of this strategy, mainly the content, the design, or the frequency in which these prompts are shown to the user (59). So, care must be taken to not overuse notifications and to ensure that the notifications are relevant and personalized. Additionally, feedback is a strategy that is perceived as important and it is provided in various forms, including visual displays (e.g., graphs), report summaries, and messages.

Goal setting is one of the most used behavior change techniques in both face-to-face and digital health interventions as well as interactive content using different types of multimedia. Social support features motivate users by leveraging social influence (e.g., peer to peer communication). Offering rewards like points or badges in exchange for executing tasks or accepting challenges, is a common approach that incentivizes target behaviors. Mobile apps using gamification can be useful for well-being and mental health interventions and may enhance motivation and reduce attrition (60).

Some studies mention app personalization and customization as being important engagement factors; aesthetics also increases the friendliness of the design since a lack of aesthetics and text-heavy presentations may bore users. However, user experience does not predict sustained engagement with mental health apps (16). Likewise, usability is best understood as a potential barrier to engagement rather than something that increases engagement on its own. Usability is particularly important in digital health interventions for conditions in which users’ abilities may differ from the general population and the specific context where implementation is occurring, e.g., within a healthcare organization or directly to users.

On the other hand, there are factors that negatively influence user engagement, increasing the dropout of users such as the difficulty to use the technology, the value and usefulness perceived by the user or the adoption rate by population outside of studies (61). A person-centered approach or participatory design is needed to identify those factors, personal or app-specific, that may aid engagement at different stages. To produce an engaging intervention, it is critical to co-create and validate features with the people who will use them. Wang and Qui (62) presented in a systematic review the influencing factors of acceptance and use behavior of mobile health applications. They say that users’ behavior is influenced by individual demographic characteristics, app design, functionality, perceived ease of use and usefulness, security, cost, and personal motivations. Social attributes, source credibility, and legal issues also affect user behavior. Our results agree with these, insofar as in our scoping review some demographic factors were perceived as influencing adherence such as age, gender, education level and socioeconomic status. Younger people, women, more literate, and with higher socioeconomic status seem to show higher levels of involvement in this type of technological solutions.

Other factors also may play a role in influencing engagement: health status, data privacy and security, involvement in design process, incentives for participation, app usage fitting in the user routines, initial training, and constant technical support.

Regarding illness severity, some may argue that apps usage could be counterproductive or harmful on illness acute phases. SlowMo, one of the apps included in this review, specifically focused on addressing persistent paranoia which is common among patients with psychosis. This app is a digital intervention integrated with eight individual CBT sessions. The results showed lower levels of paranoia however these effects did not remain over time (43).

A faulty app that presents errors or bugs to the user can also have a negative impact in user engagement (63). In line with previous findings, the existence of human support is a critical aspect of digital interventions and reinforces the importance of the therapeutic alliance even in digital interventions (64, 65). Also, credibility can lead to more engaged users if the app incorporates expertise or clinician encouragement (21). A systematic review and thematic analysis found that personalization, reinforcement, and communication (communication can also be called social support and social network) are the design features mentioned the most often (66). Another systematic review (57) studied the barriers and facilitators of user engagement strategies with digital mental health interventions. The authors presented three categories—user-related factors, program-related factors, and factors related to the technology and implementation environment—and stated that by understanding these factors, targeted strategies can be used to overcome potential obstacles to the efficient use of digital interventions.

Regarding how to assess user engagement, there is high heterogeneity of criteria and measures used. This lack of consensus makes it difficult to compare results across studies and hinders understanding of what makes apps engaging for different users (18). It is also notable that interventions are generally described as being acceptable and feasible. Several methods to measure engagement were identified, including qualitative measures (semi-structured interviews, focus groups), self-report questionnaires, ecological momentary assessments, system usage data, sensor data, and clinical measures, which are consistent with those in previous literature (67). These comprise reports of the subjective user experience, elicited by qualitative methods or questionnaires (focusing on satisfaction questionnaires, interviews about usability, etc.), and objective measures of technology usage, user behavior, and users’ reactions to the intervention (focusing on usage frequency, response to prompts, trial retention). Self-reported measures are predominant in user engagement research and target subjective user engagement by way of users’ perception of technology mainly through post experience questionnaires. In addition, there are some validated instruments that can be used for these tools, specifically for research, for instance MARS—a framework that evaluates mobile health app quality along dimensions of engagement, functionality, aesthetics, and information quality (68). The engagement scale assesses how interactive and interesting the app is, the functionality scale assesses the app's functioning and ease of use, the aesthetics scale assesses overall visual appeal and stylistic consistency, and the information subscale assesses the quality of the content. Averaged together, the four subscales indicate the MARS total score, which measures overall app quality. None of the studies included in this scoping review used MARS. For real-world implementation, it is important to include in app features to automatically assess engagement and allow adjustments. It is also necessary to improve how stakeholders measure the impact of modifiable variables on engagement to understand the magnitude of effects (54).

O’Brien and colleagues (10) proposed an approach that could better meet users’ needs. In their perspective, design of apps would ideally be corroborative, outcome oriented, process based, and expert driven. In our perspective, app developers and mHealth service providers should use this study findings to include patients’ requirements in the app-development process.

Nevertheless, mobile health technology evaluation includes several dimensions, and user engagement is only one of them. The WHO mHealth Technical Evidence Review Group developed the mHealth evidence reporting and assessment (mERA) checklist. mERA aims to assist authors in reporting mHealth-research and the mHealth intervention (69) and this is very pertinent because there is a need for mHealth developers to follow guidelines for the buildup of mHealth apps, according to usefulness, quality, and safety standards. The American Psychiatric Association designed an evaluation hierarchal framework to guide informed decision making around the use of smartphone apps in clinical care (70), in which engagement is also included. This shared discussion among all stakeholders can also promote a more informed and sustained use of mobile applications in mental health.

## Conclusion

Despite the potential benefits of mobile apps for people with schizophrenia, many interventions fail to engage their users effectively. Hence, our aim is to identify strategies and factors that may influence user engagement and facilitate prolonged use of apps for people with schizophrenia to better manage their condition. Nonetheless, there are some limitations in our study. The search was limited to articles published in English, as such, some articles may have been missed. In addition, the scoping review was not restricted to studies with high rigor, thus some of the report findings require replication since some studies lacked detail and this along with the variability in assessment tools made it challenging to summarize across studies. Because of this, a unified catalog to create a successful user engagement strategy was not achievable, which is something that is already clearer for other medical conditions. Also, there are apps which were not included in this scoping review because it did not have associated data.

Nonetheless, this scoping review provides some insights for increasing effective engagement in mobile apps for people with schizophrenia, analyzing not only user engagement strategies but also user-related factors, technology-related factors, and environment-related factors. Researchers, as well as practitioners, can use this knowledge to inform evaluations and development of new digital tools to support mental health services.

Future work should continue to explore innovative intervention strategies by which apps can support mental health in ways that appeal to people with schizophrenia, since a key component of the effectiveness of mental health apps is user engagement.
